# Association of climatic factors with infectious diseases in the Arctic and subarctic region – a systematic review

**DOI:** 10.3402/gha.v7.24161

**Published:** 2014-07-01

**Authors:** Christina Hedlund, Yulia Blomstedt, Barbara Schumann

**Affiliations:** Department of Public Health and Clinical Medicine, Centre for Global Health Research, Umeå University, Umeå, Sweden

**Keywords:** infectious diseases, climatic factors, Arctic, subarctic region, systematic reviews

## Abstract

**Background:**

The Arctic and subarctic area are likely to be highly affected by climate change, with possible impacts on human health due to effects on food security and infectious diseases.

**Objectives:**

To investigate the evidence for an association between climatic factors and infectious diseases, and to identify the most climate-sensitive diseases and vulnerable populations in the Arctic and subarctic region.

**Methods:**

A systematic review was conducted. A search was made in PubMed, with the last update in May 2013. Inclusion criteria included human cases of infectious disease as outcome, climate or weather factor as exposure, and Arctic or subarctic areas as study origin. Narrative reviews, case reports, and projection studies were excluded. Abstracts and selected full texts were read and evaluated by two independent readers. A data collection sheet and an adjusted version of the SIGN methodology checklist were used to assess the quality grade of each article.

**Results:**

In total, 1953 abstracts were initially found, of which finally 29 articles were included. Almost half of the studies were carried out in Canada (*n*=14), the rest from Sweden (*n*=6), Finland (*n*=4), Norway (*n*=2), Russia (*n*=2), and Alaska, US (*n*=1). Articles were analyzed by disease group: food- and waterborne diseases, vector-borne diseases, airborne viral- and airborne bacterial diseases. Strong evidence was found in our review for an association between climatic factors and food- and waterborne diseases. The scientific evidence for a link between climate and specific vector- and rodent-borne diseases was weak due to that only a few diseases being addressed in more than one publication, although several articles were of very high quality. Air temperature and humidity seem to be important climatic factors to investigate further for viral- and bacterial airborne diseases, but from our results no conclusion about a causal relationship could be drawn.

**Conclusions:**

More studies of high quality are needed to investigate the adverse health impacts of weather and climatic factors in the Arctic and subarctic region. No studies from Greenland or Iceland were found, and only a few from Siberia and Alaska. Disease and syndromic surveillance should be part of climate change adaptation measures in the Arctic and subarctic regions, with monitoring of extreme weather events known to pose a risk for certain infectious diseases implemented at the community level.

Climatic conditions such as ambient temperature, precipitation, and humidity are critical for the transmission of several infectious diseases. Infectious disease incidences are therefore likely to be affected by climate change. Due to the complexity of infectious disease epidemiology, and the importance not only of climatic factors but also of socio-economic and environmental factors, there are still major challenges and questions in this research area, one concerning the possible effects of climate change in the Arctic and subarctic area on infectious diseases.

The Intergovernmental Panel on Climate Change (IPCC) states that ‘average Arctic temperatures have increased at almost twice the average global rate in the past 100 years’ (1). ‘In the most northern subarctic areas the warming of the climate is expected to be the most pronounced during the winter, with higher levels of precipitation during the whole year, and with higher risk of extreme levels of precipitation’ (1). According to the IPCC report 2007, ‘the Arctic is one of the regions likely to be especially affected by climate change, because of the impacts of high rates of projected warming on natural systems and communities’ (1). The impact these projected increases in temperature and precipitation levels could have on human health are not yet fully known. However, implications for food security, increased incidences of waterborne diseases and changing habitats of vector-borne diseases have been suggested (2). The population of the Arctic regions is 4 million people, spread over a large geographical area, in an ecosystem highly adapted to a harsh and sensitive climate. Many of the indigenous populations live and work in very remote areas, with limited access to health care, and in close connection to the natural environment. Insufficient infrastructure and crowded living conditions increase the risk for the spread of infectious diseases. Together with the dependence on nature this makes the population in the Arctic especially vulnerable to climate change (2). To detect early changes in infectious diseases epidemiology in this area, it is important that research reaches over borders, since the population is spread over such large geographical areas.

In order to be able to predict the effects of climate change on the occurrence of infectious diseases in the Arctic, we first need to increase our knowledge about the relationship between climatic variability and infectious diseases. Weather is the daily state of the atmosphere, whether it is hot or cold, dry or humid. Climate is usually defined as the average weather over a certain time period. ‘Climate variability refers to variations in the mean state and other statistics (such as standard deviations, the occurrence of extremes, etc.) of the climate on longer temporal and spatial scales beyond that of individual weather events’ (3). Finally, climate change refers to ‘a statistically significant variation in either the mean state of the climate or in its variability, persisting for an extended period (typically decades or longer)’ (3).

The possible effects that climate change could have on infectious disease epidemiology in the Arctic and subarctic areas in the future have earlier been discussed (4–6), but there is no systematic review to our knowledge that has been done regarding climatic factors and infectious diseases in the Arctic. A systematic analysis of findings in this rather young and very complex research area is important in order to identify research gaps, and to evaluate the quality, strengths and limitations of the current evidence in order to see what can be learnt for further studies. We conducted a systematic review about the association between climatic factors (including both short- and long-term variations) and the occurrence of infectious diseases among humans in the Arctic and parts of the subarctic region. Our objectives were to investigate the evidence for an association between climatic factors and infectious diseases, and, if possible, to identify the most climate-sensitive diseases and vulnerable populations in the Arctic/subarctic region.

## Methods

We conducted a systematic review by analyzing studies that were published in PubMed-listed journals. Studies were included if human cases of infectious diseases were the outcome, excluding animal, vector, or vaccine studies in order to restrict the scope of the review. Although studies on the association of climate/weather and infectious diseases in animals might be relevant also regarding the health risks in humans, the link is not straightforward. An increase in pathogen prevalence in animals and vectors does not necessarily imply higher incidences in humans, and that is what we were interested in with this review. Only studies with climatic factors as exposures were included, but not studies addressing only seasonality. Seasonality studies were excluded because we were interested in impacts of meteorological factors that might change in the course of climate change. Seasonality (i.e. winter, summer, etc.) are of course associated with temperature and precipitation, but their impact on health also has other causes. Studies concerning changes in seasonality patterns would have been included, but no such study was found. Geographical areas included were Canada, Greenland (the rest of Denmark was excluded), Iceland, Norway, Sweden, Finland, Alaska, and the northern parts of Russia. The inclusions of these countries were based on the fact that the countries or areas had an Arctic or subarctic climate. The most southern parts of Russia were excluded, due to that the climate was not considered subarctic. Narrative reviews, case reports, and prediction studies were excluded, and only original studies were included.

A PubMed search was conducted in June 2011, with updates in November 2012 and May 2013. There were no publication date limits for the search. No language restrictions were made. The MESH terms used were bacterial infections and mycoses, parasitic diseases, virus diseases, climatic processes, climate, climatic, weather, temperature, precipitation, Scandinavia, Finland, Iceland, Russia, Siberia, Arctic regions, Greenland, Canada, and Alaska.

Retrieved titles and abstracts were selected by two independent readers (CH, BS) based on the criteria described above. Consensus was obtained by discussion in case of opposing decisions. The full texts of selected abstracts were read by two researchers (CH, BS), and decisions about inclusion were taken after a consensus discussion.

For each included study, two independent readers (CH, BS) filled in a data collection sheet, with information about study place, time and study population, objectives, exposures, outcomes, statistical methods, and results. An adjusted version of the SIGN methodology checklist (7) was used in order to assess the level of quality of the studies (8), including how well the study was done in order to minimize the risk of bias and to establish a causal relationship between exposure and effect.

The final grade indicating the study quality as a result of this systematic evaluation was based on the quality of data sources and methodology used, the risk of bias, and the consideration of co-factors. After consensus discussion a decision about the quality grade was taken for each included article. The same procedure was followed for the Russian articles, with discussion and evaluation by triangulation, led by a Russian-speaking researcher (CH, YB, BS).

Since there were very few articles concerning each infectious disease, the articles were summarized and grouped mainly by transmission way: food- and waterborne diseases, vector- and rodent-borne diseases, airborne diseases (viral and bacterial).

## Results

### Study selection

**Fig. 1:**
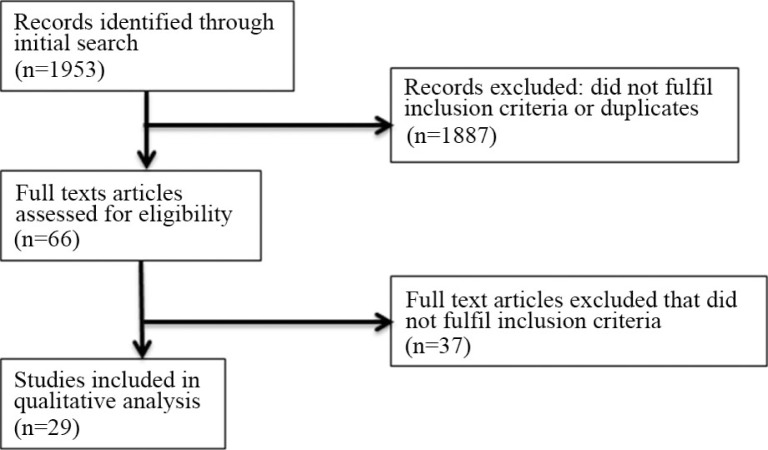
Flow diagram of study selection process (Systematic review of association of climatic factors with infectious diseases in the Arctic and subarctic region).

**Fig. 2:**
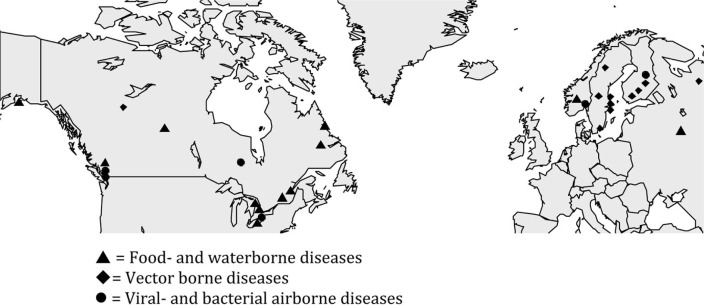
Geographical distribution of included articles on climatic factors and infectious diseases in the Arctic and subarctic area.

Initially, 1953 abstracts were found in the PubMed search. Of these, 1887 were excluded according to the predefined in/exclusion criteria. Sixty-six abstracts fulfilled the inclusion criteria and were read as full texts. Finally, 29 of these fulfilled the inclusion criteria, while the other 37 were excluded (Figs. 1 and 2).

### Study characteristics

Of the 29 included studies, almost half were made in Canada (14 studies). There was one study from Alaska, six from Sweden, four from Finland, two from Norway and Russia, respectively. There were no studies from Greenland or Iceland.

Twelve articles concerned food- and waterborne diseases and 11 articles vector-borne diseases. Three articles concerned airborne or droplet-borne viral diseases and three articles airborne bacterial diseases.

The majority of studies were ecological designs with data aggregated on a population level. There were four descriptive studies without statistical analyses, 12 time series analyses using aggregated data, and two case-crossover analyses as well as one prospective cohort study based on individual data. The main statistical methods used were regression models (Poisson, negative binomial, least square) and correlation analysis.

Two articles concerning historical malaria occurrence in Finland had the longest time span, 210 and 70 years, respectively. Seven articles had a study period of 23–38 years, three articles 13–21 years, and seven articles 6–10 years, respectively. There were eight articles with 1–5 years study period and two articles discussing one single outbreak.

The main climatic factors investigated were air temperature or precipitation. Other investigated climatic factors were humidity, snow depth, and NAO (North Atlantic Oscillation) index (Table 1).

**Table 1:** Characteristics of included studies on climatic factors and infectious diseases in the Arctic and subarctic area

Reference (grade)	Objective	Study design	Place, time	Exposure (climate factor)	Outcome (infectious disease)	Co-factors	Stat. methods	Results	Conclusions
Allard, 2010 (9)	Associations between temp. previous weeks and campylobacteriosis	Ecological, TSA	Canada, Montreal, 1990–2006	Mean weekly temp. lag 1–6 weeks	Campylobacteriosis (*n*= 0–27/week, mean *n*=9/week)	Seasonal and secular trends, # camp. lag 1 to 12 weeks	Negative binominal regression	Final adjusted model: IRR 1.008 (1.0025–1.0131) per 1°C increase in temp. >10°C lag 1 to 6 weeks	Increased temp. campylobacteriosis↑
Arsenault, 2011 (11)	Association between environmental characteristics and campylobacteriosis	Ecological, TSA	Canada, Quebec, 1996–2006 (climate data 1996–2003)	Seasonal temp. (mean of daily max. and min.), seasonal precip. (mean of daily max. and min.), categorized (low, average, high)	Campylobacteriosis (*n*=28,519)	Age groups, season, ruminant density, slaughter-house, poultry density	Multilevel Poisson regression (temp.+co-variables). Univariate analysis (precip.)	Precip. (Reference: lowest level). Average level IRR 1.04 (CI 0.99–1.09). High level IRR 1.11 (CI 1.02–1.20). Temp. n.s.	Increased precip. campylobacteriosis↑ Temp. n.s.
Auld, 2004 (2)	Role of excessive rainfall for one outbreak of waterborne infectious disease	Descriptive	Canada, Walkerton, 2000	Monthly and daily precip., excessive rainfall	Campylobacteriosis, *E. coli* 0157:H7	None	Descriptive	Excessive rainfall possible related to outbreak. No statistical analyses	Increased precip. campylobacteriosis↑
Sandberg, 2006 (3)	Association between campylobacteriosis and assumed biological risk factors	Ecological	Norway, 2000–2001	Average rainfall in mm/county and temp. for 2000–2001	Campylobacteriosis (*n*=2,109)	# people in the county, urbanization, kg chicken sold. # people drinking treated water. #animals in county.	Population-averaged Poisson regression (trend- and risk factor model)	Rainfall: IRR 1.006 per mm (CI 1.005, 1.007), adjusted for co-factors	Increased precip. campylobacteriosis↑
Ravel, 2010 (3)	Association between salmonellosis and human behavioral risk factors and meteorological factors	Ecological, TSA	Canada, Waterloo, 2005–2008	Mean monthly and mean daily temp. Total monthly precip.	Endemic salmonellosis cases (*n*=216)	None	Poisson regression, (univariate analysis for 14 risk factors, age and sex)	Year and mean temp. current month coeff. 0.0403, *p* <0.0001. Year and mean temp. previous month: coeff.= 0.0298, p<0.0003. Monthly precip. n.s.	Increased temp.-salmonellosis↑, precip. ns.
Fleury, 2006 (4)	Associations between weekly enteric bacterial disease and short-term variations in temperature	Ecological, TSA	Canada, Alberta and Newfoundland Labrador, 1992–2000	Mean weekly temp. Several lags for 0–6 weeks. Temp. thresholds	Salmonellosis (Alberta *n*=6,282, NF Labr. *n*=986). Campylobacteriosis (Alb. *n*=1,743, NF Labr. *N*=1188). Enteropathogenic *E. coli* (Alb. *n*=9,664)	Seasonal effects, long-term trends, vacations days. Population size/climate health zone	Generalized linear model, generalized additive model	1°C increase of temp. >−10°C threshold: Alberta, salmonellosis: RR 1.012 (CI 1.009–1.015), Camp.: RR 1.022, (CI 1.019–1.024), E. coli (threshold 0°C) RR 1.060 (CI 1.050–1.069). 1°C increase of temp. >0°C NF Labrador, Camp.: RR 1.045 (CI 1.033– 1.058)	Increased temp.-cases of bacterial diseases ↑
Thomas, 2006 (4)	Association between waterborne disease outbreaks and high impact weather events	Case-crossover design	Canada, 1975–2001	Rainfall (3 different parameters), temp. (3 parameters), stream flow (6 parameters)	One outbreak of waterborne infectious diseases (288 outbreaks, 92 included).	Seasonality	Conditional logistic regression	For rainfall events >93rd percentile, OR 2.283 (CI 1.216–4.285). For each degree-day unit increase, OR increased by 1.007	Extreme precip. ↑↑, Increased nr of warm days ↑ – Outbreak of waterborne diseases
Harper, 2011 (4)	Association between weather variables and IGI-related clinic visits	Ecological, TSA	Canada, Nain, Nunatsiavut, 2005–2008	Mean weekly temp., total water volume input (rain+snowmelt), weekly snow depth	IGI-related cases (vomiting and diarrhea) presenting to health clinics+specific exclusion criteria's (*n*=541)	Yearly trend	Univariable unconditional Poisson model. Multivariate zero inflated Poisson.	Multivariate analyses: High water input (>90th%), lag 2 weeks: IRR 1.34. p<0.04, lag 4 weeks: IRR 1.31, p<0.03. High temp. >90th%, lag 4 weeks: OR 3.91, p<0.50	Increased precip./snow IGI-related cases ↑
Teschke, 2010 (4)	Association between intestinal infectious diseases and environmental factors	Retrospective cohort study	Canada, Township of Langley, Vancouver, British Columbia, 1995–2003.	Rainfall over one- and two-week period with lag 0, 2-, 5-, and 10	ICD diagnoses 003, 004, 007, 008, 009. Intestinal infectious diseases with potential of being waterborne	Sex, age, year, season, duration of residence, water systems, drinking water disinfection, sewage disposal, land use, well depth.	Logistic regression, separate model for each lag	OR for all lags and all rain levels ns. except for lag 0: 25 to >100 mm of rainfall OR 1.15 (CI 1.05–1.27)	Increased precip. n.s.
McLaughlin, 2005 (2)	Investigation of climatic factors and one Vibrio parahemolyticus outbreak	Descriptive study	USA, Alaska, Prince William Sounds, July 2004	Mean monthly water temp. in July and August	Gastroenteritis caused by Vibrio parahemolyticus (*n*=22/189 passengers)	None	None	Unusual warm water temp. at oyster farm prior to outbreak	Water temp. >15°; risk for Vibrio p. gastroenteritis outbreak↑
Vasil'ev, 1970 (2)	Associations between dysentery, meteorological conditions and number of flies	Ecological	Russia, Moscow region, Oreko-Zuevo, 1950–1968	Average temp. in May–Sept.	Dysentery cases (Flexner, Newcastle and Sonne Dysentery)	Nr of flies	Correlation coefficient	Corr. coeff. for temp. and dysentery incidence (Flexner, Newcastle) in 1950–58: 0.84±0.1, 1959–68 −0.1±0.3. For Sonne n.s.	Increased summer temp. Flexner and Newcastle Dysentery↑
Greer, 2009 (4)	Association between environmental factors and frequency of norovirus outbreaks in the winter months	Ecological, TSA	Canada, Greater Toronto area, 2005–2008	Mean air temp. Mean total precip. Mean lake Ontario temp. Mean flow in the Don river	Outbreak of norovirus (*n*=253 outbreaks)	None	Poisson regression; case-crossover analysis (multivariate)	Case-crossover: Low lake temp. (≤4°C): HR 5.61 (CI 2.81–11.12); high river flow (>2.5 m^3^/s) HR 3.17 (CI, 2.30–4.36), with lag 1–7 days	Colder lake temp. and high river flow-risk for norovirus outbreak↑
Lindgren, 2001 (3)	Association between tick-borne encephalitis (TBE) incidences and climatic factors	Ecological	Sweden, Stockholm county, 1960–1998	Seasonal temp. current and previous year. Nr of days with low snow cover the preceding 2 years	Tick-borne encephalitis (TBE)	None	Multiple regression	*R* ^2^=0.58 *p*<0.0001. Full model: Two mild winters, temp. (8–10°C) and extended autumn (5–8°C) in previous year and temp. (5–8°C) early in incidence year	Warmer and prolonged warm seasons- TBE↑
Haemig, 2010 (22)	To predict nr of tick-borne encephalitis (TBE) cases by environmental factors the previous year	Ecological, TSA	Sweden, Stockholm and Uppsala, 1984–2008.	Mean monthly temp. and precip.	TBE (Stockholm *n*=1,286, Uppsala *n*=214)	Fox, mink population	Multi-variate linear regression	Stockholm. Dec. precip. coeff. 0.32 (*p*=0.039) adjusted for #mink. Temp n.s	Increased Dec. precip. previous year- TBE↑
Tokarevich, 2011 (2)	Associations between tick-borne encephalitis (TBE) incidence, vector distribution and climatic factors	Ecological, longitudinal	Russia, Archangelsk Oblast (AO), 1980–2009	Mean annual temp.	TBE (1980–89: *n*=16, 1990–99: *n*=207. 2000–09: *n*=697.)	None	Regression analysis (1980–2009). Correlation analysis (1990–2009).	Temp – TBE (1990–2009) *r*=0.50 (CI 0.36–0.64, *p*=0.0248)	Increased temp.- TBE↑
Palo, 2009 (4)	Association between nephropathia epidemica (NE), NAO index and bank vole population dynamics	Ecological, TSA	Sweden, northern part, 1959–1975, 1985–2006	Mean NAO index from November to march	Nephropathia epidemica	Vole mortality, trap index. Improved diagnostics accounted for by adjusted year trend.	General linear model.	Full model incl. vole factors and NAO index in current and previous years. R2 0.45 n.s.	NAO index n.s.
Pettersson, 2008 (2)	Association between a nephropathia epidemica outbreak and climatic factors	Ecological, descriptive	Sweden, 1998–2007, outbreak 2006–2007 Västerbotten County	# days with minimum snow cover. Mean temp. in Dec. 1998–2006	Nephropathia epidemica (n=488)	None	Descriptive	A warm December and with little snow cover preceded the outbreak in 2006/2007	Unusual warm winter and low snow cover preceded outbreak of NE
Ryden, 2012 (4)	Associations between environmental parameters, mosquito abundance and type B tularemia	Ecological, TSA	Sweden, Dalarna County, 1981–2007	Mean temp., humidity, total precip. for summer, spring, winter, previous fall and summer. Cold winter days (<−7.3°C), snow cover (<10 cm) preceding winter	Type B tularemia (*n*=370)	Mosquito abundance	Neg. binominal regression. Model validation with pseudo R2.	Summer temp preceding summer coeff. 0.65. Summer precipitation coeff +0.012. Cold winter and low snow coverage coeff. −0.15	Cold winter, low snow coverage↓, warm summer temp. previous year ↑ – type B tularemia
Bennet, 2006 (4)	Association between climatic factors and summer variations of Lyme borreliosis (LB)	Ecological	Sweden, Blekinge County, 1997–2002	Mean monthly temp., humidity, precip. # days with relative humidity (RH) >86%, 14 days lag. # winter days <0° current and previous year.	Lyme borreliosis ‘combination of tick bite, erythema migrans and antibiotics prescribed’. (*n*=3,437)	Gender, age	Two level multivariate Poisson regression	Temp. IRR 1.12 (CI 1.08–1.16, *p*<0.001). # Winter days with mean temp:<0° C: IRR 0.97 (CI 0.97–0.98, p<0.001). Precip.: IRR 0.92 (CI 0.84–0.99, *p*<0.05). Days with RH >86%: IRR 1.04 (CI 0.76–0.96, *p*<0.05.)	Increased temp., increased humidity ↑, Colder winter, increased precip. ↓- borreliosis
Hulden, 2005 (2)	Associations between malaria and climate (historical perspective)	Ecological	Finland, southern parts, 1800–1870	Annual, seasonal, monthly temp. current and previous year (Helsinki, Tornedalen, St Petersburg, Stockholm)	Death from Malaria (from parish registers, incl. synonyms for ‘malaria’) (*n*=5,413 malaria deaths)	None	Correlation coefficient, not specified.	Summer temp. previous year in Stockholm (1800–1870) Corr. Coeff. 0.4708	Increased temp. June-July previous year- Malaria↑
Hulden, 2009 (2)	Association between environmental and climatic factors and malaria (historical perspective)	Ecological	Finland, 1750–1960 (period 1: 1750–1830, period 2: 1830–1890, period 3 1890–1960)	Mean temp. June–July previous year	Nr of malaria cases estimated from malaria deaths (from parish registers, assuming 2% case fatality)	None	Correlation coefficient r. (Univariate analyses for three time periods)	Summer temp. Uppsala (longest time period): period 1. *r*= 0.449 (*p*<0.001), period 2 *r*=0.433 (*p*<0.001), period 3 *r*=0.297 (*p*<0.05)	Temp. n.s.
Brummer-Korvenkontio, 2002 (2)	Epidemiology of Sindbis Virus (SINV) seroprevalence and Pogosta disease (PD, also known as ‘Ockelbo disease’) in Finland	Ecological, descriptive	Finland, 1973–96	Snow depth (cm). Temp. in May–July. Daily mean temp. 10 days prior disease. (Seasonality)	SINV-seroprevalence, and PD (1973–89: 6,320 patients with ‘suspected rubella’: *n*=107 PD cases. 1981–96: 9,842 patients with ‘Suspected PD’: *n*=2,183 cases)	Sex, age	Descriptive	Graphical display indicates association of temp. and snow depth with PD incidences	Snow depth late winter and temp. May–June of importance for PD cases?
Tizard, 1976 (2)	Association between toxoplasmosis and climatic factors	Ecological	Canada, 1961–1974	Rainfall (Mean annually, monthly. Min. summer, max. annual, august temp.)	Toxoplasmosis (11,934 samples totally/11.5% positive for toxoplasmosis). Serum samples analyzed for 1/16 or 1/1024 dilution.	Age standardization of prevalences	Correlation coeff. of rain resp. temp. with% of positive samples	1/16 dilution: Mean annual rainfall *r*=−0.08057 (n.s.), average august rainfall *r*=0.399 (n.s.). 1/1024 dilution: Mean annual rainfall *r*=0.13482 (n.s.), average august rainfall 0.7069 (*p*<0.05)	Dryness in late summer previous year- toxoplasmosis↑
Orstavik 1980 (2)	Associations between Respiratory Syncytial Virus (RSV) infections and environmental/climatic factors	Ecological	Norway, Oslo, 1972–1978.	Mean monthly temp. Hours of sunshine/month. Amount of rainfall/snow/month. Average humidity/month	RSV (*n*=464)	Air pollution, SO_2_ in the air	Chi-square test, Wilcoxon–Mann–Whitney, Kendall rank correlation coeff. test	Higher temp., hours of sunshine negatively correlated with # RSV infections/month (*p*<0.05)	Colder temp. and few hours of sunshine – RSV↑
Mäkinen 2009 (3)	Association between respiratory tract infections (RTI), outdoor temp. and humidity	Longitudinal prospective cohort study	Finland, Kajaani garrison in Central Finland, 2004–2006	Mean and max temp. of the preceding 3 days and 2 weeks of disease onset. Absolute humidity (g/m^2^) 3 days prior to disease onset	Upper respiratory tract infections (URTI). Lower resp. tract infections (LRTI) (*n*=643 RTI)	None	Generalize additive model. Logistic regression. ANOVA repeated measures. Univariate analysis.	1°C decrease in temp. increased URTI risk: OR 0.96, *p*<0.0001, LRTI: OR 1.02, p 0.038. Decrease of 1 g/m^3^ in absolute humidity increased URTI risk OR 0.9, *p*<0.001, LRTI n.s.	Colder temp. and low humidity – URTI ↑
Tang, 2010 (3)	Association between influenza infections and temp., relative humidity and rainfall	Ecological, TSA	Canada, Vancouver, 2000–2007	Weekly temp., humidity and rainfall	Influenza A and B infections	Relative humidity and temp. controlled for each other	Dynamic regression model	Influenza A: Rel. humidity Lag 1 Estimate 0.209, p 0.0079. Influenza B. Mean temp: Lag 2. Estimate 0.0190, p 0.0205. Relative humidity lag 2. Estimate −0.063, p 0.0209. Influenza A-rainfall ns., temp. ns. Influenza B-rainfall n.s.	Increased humidity- Influenza A ↑, Increased humidity and temp. – Influenza B ↓
Crighton 2007 (2)	Patterns of pneumonia and influenza hospitalizations in Ontario and the factors that determine them	Ecological	Canada, Ontario, 1992–2001	Mean annual temp.	Pneumonia and influenza hospitalizations, (ICD9. 480–487), from medical chart records. (*n*=241,803)	Social and environ., behavioral, healthcare factors. Sex, age.	Ordinary least square regression	Females 65+, temp.: Regression coeff. −0.031 (SE 0.017, p 0.079). Men 65+, temp: Regr. coeff. −0.039 (SE 0.014, p 0.006). Females and men <65 years n.s.	Increased temp. – pneumonia hospitalizations↓
Ng, 2008 (4)	Association between environmental factors and legionellosis	Ecological, case-crossover design, TSA	Canada, Toronto, 1978–2006.	Temp., precip., atmospheric pressure, relative humidity, hydrological data for lake Ontario	Legionellosis	Seasonality	Negative binominal regression, case-crossover design	Univariate: Mean rainfall IRR 0.954 (CI 0.941–0.967, *p*<0.001), mean humidity IRR 1.005 (CI 1.000–1.010, *p*=0.034). Multivariate: Rainfall ns., humidity IRR 1.004 (CI 1.029–1.059, *p*<0.001)	Temp., precip., humidity ns. Changes in local watershed of importance for legionellosis
Dodek, 2011 (3)	Association of ICU admissions for community acquired pneumonia (CAP) with temp. and precipitation	Ecological, TSA	Canada, Vancouver, 2002–2006	Weekly mean temp., range temp., total precip.	CAP from ICU database (740 cases)	Weekly influenza-like illness, seasonality	Poisson regression (univariate and multivariate)	Univariate: Weekly average, temp. range and total precip. n.s. Multivariate: average temp: RR 0.99 (0.98–1.01). temp range: RR 1.01 (0.96–1.06). precipitation: RR 1.00 (0.98–1.02)	Temp. and precip. lag 2–3 weeks n.s.

TEMP.=temperature; Precip.=precipitation; IRR=incidence relative risk; RR=relative risk; OR=odds ratio; HR=hazard ratio; CI=confidence interval; n.s.=not significant; TSA=time-series analysis; Coeff.=coefficient; Camp.=campylobacter; IGI-related=infectious gastrointestinal illness-related; TBE=tick-borne encephalitis; NE=nephropathia epidemica (‘Vole fever’); SINV=Sindbis virus (causative agent for PD disease); PD=Pogosta disease, also called Ockelbo disease; RSV=respiratory syncytial virus; ICU admissions=intensive care unit admissions; *n*=numbers; #=number of.

## Food- and waterborne diseases

**Allard** (9) investigated the relationship between temperature and **campylobacteriosis** incidence in Montreal, Canada, January 1990–March 2006. From their results, two distinct transmission routes for campylobacter were suggested, one influenced by temperature increases in the previous 6 weeks while the other transmission route was not associated with temperature. The possible effects of climate change were discussed, suggesting that an increased annual temperature in Montreal to 4.5°C by 2055 could mean a 23% increase of campylobacteriosis cases (Grade 5).

**Auld** (10) investigated the relationship between precipitation and an outbreak of **campylobacteriosis** and **E. Coli 0157:H7** in Walkerton Canada (9–12 May 2000). They suggested that the significant accumulation of rainfall amount over days and ground saturation had increased the risk for the contamination of groundwater leading to the outbreak. No statistical analyses or descriptive study of one outbreak (Grade 2).

**Arsenault** (11) investigated the association between environmental characteristics and **campylobacteriosis** cases in Quebec, Canada, 1996–2006. They found that the incidence of campylobacteriosis was significantly reduced in municipalities with low levels of total precipitation. No significant association with temperature was seen (Grade 4).

**Sandberg** (12) investigated the association between several biological risk factors and **campylobacteriosis** in Norway between 2000 and 2001. They found an association between higher rainfall and campylobacteriosis incidence. Several relevant co-factors were included, but on the other hand only median climatic values for larger geographic areas were used, possibly leading to an underestimated effect of local precipitation or temperature (Grade 3).

**Ravel** (13) investigated the association between **salmonellosis** cases, human behavioral risk factors, retail chicken meat contamination and meteorological factors in Waterloo, Canada, from June 2005 until May 2008. Monthly distribution of salmonellosis cases was associated with ambient temperature, with a significant seasonal peak in June and July, but not with precipitation. A univariate, but no multivariate, analysis was made for several factors (Grade 3).

**Fleury** (14) investigated the association between weekly **enteric bacterial diseases** and short-term variations in temperature in Alberta and Newfoundland, Canada, 1992–2000. Average weekly temperature from 0 to 6 weeks prior to the incidence of weekly disease was found to be significantly associated with all tested pathogens in each location, also when controlled for seasonal and long-term trends (Grade 4).

**Thomas** (15) investigated the association between **waterborne diseases outbreaks** and high impact weather events by analyzing 92 outbreaks (‘cases’) of waterborne diseases linked to a drinking water supply in Canada between 1975 and 2001. The number of maximum degree-days above 0 degrees and extreme rainfall percentile were associated with outbreak risk, with a doubled risk when there was extreme rainfall. Because of the long study period, controlling for demographic changes would have been valuable. Climatic parameters and outbreaks (cases) were well defined (Grade 4).

**Harper** (16) investigated associations between weather variables and water quality, as well as weather variables and infectious gastrointestinal illness (**IGI-related) clinical visits** in Nain and Nunatsiavut, Canada, between 2005 and 2008. A significant association between lagged weekly water volume input (rain and snow) and IGI-related clinical visits was seen, also when controlled for other climate parameters and yearly trends. Due to the small village size of around 1,000 persons, statistical power could be a problem (Grade 4).

**Teschke** (17) investigated the relationship between the incidence of **intestinal infectious diseases** and environmental factors potentially contributing to drinking water quality in Canada 1995–2003. A large cohort was followed during these years and crude rates suggested increased physical visits when rainfall increased, but this trend was not significant after controlling for other variables (Grade 4).

**McLaughlin** (18) investigated a gastroenteritis outbreak caused by **Vibrio parahemolyticus** in Prince William Sounds, Alaska, US, in July 2004. The article includes a retrospective cohort study, an outbreak investigation report, and a description of active surveillance performed. For this review, only the descriptive part of the article concerning the association between water temperature and the outbreak was taken into account. Water temperature above 15°C at the time of oyster harvest was suggested as an appropriate threshold and risk indicator of Vibrio parahemolyticus gastroenteritis based on biological plausibility and relationship in time (Grade 2).

**Vasil'ev** (19) investigated the associations of incidence of **dysentery with** meteorological conditions and the number of flies in the Moscow region of Oreko-Zuevo, Russia, 1950–1968. A correlation between summer temperature and Flexner and Newcastle dysentery with the number of flies as intermediate factors was seen, but not for Zonne dysentery. No co-factors were included, and the correlation coefficient that was used gave an imprecise effect estimate (Grade 2).

**Greer** (20) investigated the relationship between local watershed conditions and **Norovirus outbreak** risk in the Greater Toronto area, Canada, 2005–2008. Colder lake temperature and increased river flow were associated with an increased outbreak risk, suggesting a source-water reservoir for Norovirus. The hypothesis was that this reservoir might be maintained through the discharge of wastewater containing virus particles; wintertime seasonality may be explained by enhanced viral persistence at low temperatures. Multivariate analyses were performed (Grade 4).

## Vector- and rodent-borne diseases

**Lindgren** (21) investigated the association between **tick-borne encephalitis (TBE)** incidence 1960–98 and climatic factors in Stockholm County, Sweden. An increased TBE incidence was related to two mild consecutive winter seasons, temperature favoring spring development and extended autumn activity in the last year. The complex statistical model included several biologically plausible factors (Grade 3).

**Haemig** (22) attempted to developed models that used data for mean monthly temperature and precipitation, as well as data for fox or minx abundance, from one year to predict the number of human **TBE** cases the following year in Stockholm and Uppsala, Sweden, 1984–2008. A correlation between December precipitation and TBE incidence was seen, but since no other months showed a significant correlation the relationship might be by chance (Grade 3).

**Tokarevich** (23) investigated the relationship between the mean annual air temperature and the **TBE** incidences in Arkhangelsk Oblast, Russia 1980–2009. The observed increases in TBE incidences were related to the expansion of the vectors habitat, suggested to be partly caused by climate change. No co-factors were investigated and the statistical part and results not clearly explained. Despite these limitations, the large correlation coefficient between temperature and TBE incidences indicates a possible relationship (Grade 2).

**Palo** (24) used time series analyses to investigate the association between **Nephropathia epidemica (NE)**, winter NAO index (North Atlantic Oscillation index, used as a proxy for climate variability), and bank vole population dynamics in northern Sweden, 1959–1975 and 1985–2006. The abundance of bank voles was found to be the most important factor for NE, and an association with the NAO index was not found. Two different longer study periods were included, controlling for long-term trends, but not investigating the effect of single weather variables such as temperature or precipitation (Grade 4).

**Pettersson** (25) analyzed the large outbreak of **Nephropathia epidemica** in Västerbotten, northern Sweden, in 2006–2007 (488 cases) and discussed the possible importance of climatic factors for the outbreak. The annually number of NE cases in 1998–2007, the number of days with minimum snow cover per year and the average temperature in December during these years were investigated. No statistical analyses were made, but due to the biological plausibility and relationship in time, the warm temperature and extreme thin snow cover in December 2006 were suggested to have contributed to the outbreak of NE in 2007 (Grade 2).

**Ryden** (26) developed a statistical model to investigate associations between environmental parameters, mosquito abundance and **type B tularemia** in Sweden 1981–2007. A cold winter and low snow coverage was associated with fewer tularemia cases, while warm summer temperatures in the preceding year were associated with an increased number of cases. The main conclusion was the importance of mosquitoes for tularemia transmission in boreal forest regions. The statistical model was thoroughly developed with several relevant co-factors included (Grade 4).

**Bennet** (27) investigated the association between climatic factors and **borreliosis** cases in Blekinge County, southern Sweden, 1997–2002. Increased monthly mean summer temperature and the number of summer days with relative humidity above 86% were both associated with an increased number of borreliosis cases, while more cold winter days below zero degrees and increased monthly mean summer precipitation were both associated with fewer borreliosis cases. Changes in human outdoor behavior were suggested to be mediating these associations. Several climate variables, gender, and age were included in multivariate analyses (Grade 4).

**Hulden** (28) investigated the relationship between temperature and **malaria** in Finland 1800–1870. The temperature in June and July in the previous year was associated with the number of malaria deaths in all four cities included. No co-factors were included, and due to the reliance on historical records, the data validity contains some uncertainties (Grade 2). **Hulden** (29) investigated in a later study showed the significance of different factors assumed to affect **malaria** trends in Finland, 1750–1960. The average household size and consolidation of land by redistribution were highly correlated with the decline of malaria cases, while neither the vector nor the temperature had an impact of the long-term trend. Univariate analyses and correlation coefficient was used, with no effect size (Grade 2).

**Tizard** (30) analyzed the prevalence of **toxoplasmosis** in 11,000 serum samples and their correlation with precipitation and temperature in 10 Canadian cities, 1961–1974. A significant correlation coefficient between the prevalence of antibodies to toxoplasma and dryness during late summer was reported. A source for climate data was lacking, selection bias of serum samples likely and no co-factors were included (Grade 2).

**Brummer-Korvenkontio** (31) aimed to describe the appearance of **Sindbis Virus infections (SINV)**, the incidence of **Pogosta disease (PD**, caused by SINV) and the association between PD and climatic factors in Finland 1973–1996. The depth of snow cover in late winter and temperature May–July seemed to be associated with PD cases. No co-factors were taken into account in this descriptive study, and a high risk of bias seemed likely in the overall method (Grade 2).

## Airborne diseases

### Airborne viral diseases

**Orstavik** (32) investigated the association between climatic factors and Respiratory Syncytial Virus **(RSV)** infections in Oslo, Norway 1972–1978. A negative correlation was found between mean daily temperature per month, hours of sunshine per month, and the monthly occurrence of RSV infections. No source for climate data was reported and univariate analysis with correlation coefficient was used (Grade 2).

**Mäkinen** (33) investigated the relationship between numbers of **respiratory tracts infections** and mean and max temp of the preceding 3 days and 2 weeks of disease onset, as well as absolute humidity (g/m^2^) 3 days prior to disease onset. The defined study population was young men in the Kajaani garrison in northern Finland, July 2004–January 2006. Cold temperature and low humidity were associated with increased occurrence of URTI. The relationship between LTRI and temperature was non-linear, with the highest risk between 0 and 10 degrees. No co-factors were included (Grade 3).

**Tang** (34) investigated the association between weekly temperature, humidity and rainfall and incidence of **Influenza A and B** infections in Vancouver, Canada, 2000–2007, and also in several other cities outside the Arctic region. Increased humidity was in multivariate analysis associated with an increased number of influenza A cases. Increased humidity and increased mean temperature were instead associated with a decreasing number of influenza B cases (Grade 3).

### Airborne bacterial diseases

**Crighton** (35) investigated the relationship between mean annual temperature and **pneumonia and influenza hospitalizations** in Ontario, Canada, 1992–2001. An increased temperature was associated with a decreased number of pneumonia cases among both men and women over 65 years. Effects of cold climate on the immune system's resistance to respiratory infections among elderly, and poor housing were discussed as possible explanatory and intermediate factors. The multivariate model did not include temperature, seasonal effects were not taken into account, and risk factors other than climatic factors were in focus in this article (Grade 2).

**Ng** (36) investigated the association between temperature, precipitation, atmospheric pressure, relative humidity and hydrological data for Lake Ontario and **legionellosis** cases in Toronto, Canada, 1978–2006. After adjusting for seasonal effects, changes in the local watershed, based on the hydrological data for Lake Ontario, rather than changes in weather, were highly associated with Legionella. Multivariate analysis of several climatic factors as well as additional statistical testing for other regions with same result was done (Grade 4).

**Dodek** (37) investigated the relationship between several climatic factors and the number of ICU admissions for **CAP (community acquired pneumonia)** in Vancouver, Canada, 2002–2006. A seasonal local variation was found, but this was not significantly related to antecedent weather conditions. Some co-factors were included, and sound methods were used (Grade 3).

## Discussion

We conducted a systematic review to investigate the evidence for associations between climatic factors and infectious diseases in the Arctic and Subarctic area, and to identify the most climate-sensitive diseases and populations most vulnerable for adverse effects of climate change.

Most of the included studies had a study period of 6–38 years, two studies had a study period longer than 70 years, and the rest had a short study period of less than 5 years. A long time span with valid climate- and disease-data over time is needed in order to study climate change effects on infectious disease incidences. Therefore, in our review the majority of the articles concerned the association between infectious diseases and weather or climatic variability.

There was mostly a multidisciplinary research team covering all the fields of infectious disease medicine, climatology and statistics that had written the articles with the highest quality grade. Valid climate data and infectious disease data have been used in the majority of the studies. The quality would have been rated higher in several articles if multivariate analyses including co-factors had been done.

The majority of the included studies had aggregated data on a population level; therefore, it is difficult to draw conclusions regarding which populations are most vulnerable for climatic factors. Overall, it is likely that the most vulnerable populations are likely those living in remote areas where adaptation to climatic changes is most difficult, for example due to insufficient economical support or lacking infrastructure. An overall need for more research in several of the circumpolar countries was seen for all disease groups. There were no studies from Greenland or Siberia; very few from Alaska and Russia, and more articles are needed from the most northern parts of Scandinavia.

### Food- and waterborne diseases

Food-borne diseases such as salmonellosis and campylobacter are likely to be affected by higher temperature due to an increased growth, survival and replication of food-borne pathogens.

The two major pathways for waterborne diseases such as cryptosporidiosis, giardiasis and calici virus infections are often divided into drinking water and recreational water sources. Floods, extreme precipitation and storm surges are examples of climatic factors that are likely to increase the risk for waterborne infections through bacterial contamination of water sources. In the same way as for food-borne pathogens, warmer temperatures can also increase the growth of waterborne pathogens.

The majority of the articles concerning food-and waterborne diseases were made in Canada, one from Russia, Norway and Alaska, respectively. Since the geographical area of Canada spans over several climatic zones and the studies were done in different parts of the country, including rural villages, the study results could be representative for other parts of Arctic with similar climate and socioeconomic conditions.

Several of the articles were time series analyses with univariate analyses for one climate factor (mainly temperature or precipitation) and one food- and waterborne disease, most often campylobacter. Two articles concerned climatic factors other than temperature and precipitation – water temperature at the time of oyster harvest and hydrological data such as lake temperature and river flow (18, 36). Many of these articles included a high number of cases and valid climate and disease data, with sufficient statistical power.

In our review, strong evidence for an association with climatic factors was found for food- and waterborne diseases. Increased temperature was the weather factor most frequently associated with increased incidences of food- and waterborne diseases. This association was seen in five articles of varying quality (Grade 2–4). In one article of high quality (11), there was no significant association between campylobacteriosis and temperature.

These findings are consistent with studies from Europe and Australia where increased temperature were shown to be associated with a higher salmonella incidence (38–40). The risk of obtaining campylobacteriosis has also been associated with an increased weekly temperature, in Europe, Canada, Australia and New Zealand (39). Both salmonellosis and campylobacteriosis have also been shown to decrease in number of cases after implementation of food safety regulations (40). Therefore, this is thought to be one of the most important adaptation measures in order to prevent food-borne diseases in a warmer Arctic.

Increased precipitation was associated with food- and waterborne diseases in several articles. In two articles no significant association could be found. One of these articles (13) concerned salmonellosis, which was only associated with temperature, but not precipitation. In the other article (17), no association was found between intestinal infectious diseases and precipitation, possibly due to over-adjustment.

The association between increased or extreme precipitation and waterborne diseases in the Arctic that was seen in our review is consistent with the findings in a recent systematic review that investigated the relationship between extreme water-related events and waterborne diseases globally (41).

The study by Harper showed a valuable way to perform syndrome surveillance of food-and waterborne disease in a resource poor local setting (16). The study was done in a rural village in northern Canada at high risk of being affected by climate change due to the geographic location and insufficient infrastructure, which could make laboratory test based surveillance difficult. In this study, IGI-related clinical visits were the major outcome, instead of specific diagnoses for different food- or waterborne diseases. The article by Thomas was a good example of how to systematically analyze the association between climatic factors and waterborne outbreaks on a national level (15). This kind of study is valuable as part of a scientific background in the process of making national adaptation plans for handling extreme climatic events and waterborne outbreaks.

### Vector- and rodent-borne diseases

The incidence of vector-borne diseases, for example TBE and borreliosis, as well as rodent-borne diseases (for example Nephropathia Epidemica), are likely to be influenced by climatic factors such as temperature, precipitation and changes in length of seasons, due to the effect these factors could have for habitat suitability, reproduction rates, distribution and abundance of vectors and rodents. The transmission pathways for vector and rodent-borne diseases are often very complex, with several possible co-factors such as, for example, human behavior, vector abundance, and land use.

Only a few diseases were addressed in more than one publication. Therefore, the scientific evidence for a link between climate and specific vector- and rodent-borne diseases is weak, although several articles were of very high quality.

An increased temperature seemed to be of importance for several of the vector-borne diseases, specifically warmer seasons, longer summer and shorter winter season. The amount of precipitation and the depth of snow cover also seemed to play a role for several of these diseases. Thus, increased TBE incidence was associated with mild winters, extended autumn periods in the previous year and higher temperatures (5–8°C) in the same year (21), increased December precipitation (22) and increased air temperature (23). A large outbreak of NE in northern Sweden was suggested be related to a high average temperature in December the preceding year and a thin snow cover, but the study lacks statistical confirmation (25). In another other article about NE, no significant association with climatic factors (North Atlantic Oscillation index) was seen (Grade 4). Due to this, no safe conclusions can be drawn about the association between NE and climatic factors from this review. A sophisticated statistical model with several relevant co-factors included was made for tularemia, showing an association between a cold winter, low snow coverage and a decrease of tularemia cases (26). Increased monthly mean temperature and higher humidity were associated with an increased number of borreliosis cases, while more cold winter days and increased precipitation were associated with fewer cases (27). An increased number of Malaria cases was associated with increased temperature in June-July the previous year (28), incidence of toxoplasmosis was associated to dryness in late summer (30), and incidence of Pogosta disease was associated to an increased depth of snow cover in late winter and increased temperature May–July (31). These three articles concerning malaria, toxoplasmosis and Pogosta disease were all of lower quality (Grade 2), which makes the associations shown uncertain.

The results in our review concerning vector-borne diseases in the Arctic are similar to the ones in an earlier review regarding vector-borne diseases and climate on a global level (42). Increased surveillance of both vector- and rodent-borne diseases, as well as the vectors and rodents themselves, is essential in order to discover changes in habitats and incidences, and to assess the importance of climatic factors for these changes. Information made available to the public in order to increase awareness and knowledge about these diseases seems to be one of the best adaptation measures.

### Viral- and bacterial airborne diseases

Airborne viral diseases such as RSV, influenza and airborne bacterial diseases such as pneumonia are usually transmitted by airborne droplets from person to person. When being exposed to these pathogens, the individual immune defense plays a major role as to whether the person falls ill, or if they can resist the disease. It has been suggested that climatic factors could play a role both for the airborne transmission of viral and bacterial pathogens into the respiratory tract and also for the immune status among different persons (43).

There were three articles from Canada, concerning bacterial airborne diseases (pneumonia, legionella and ICU admissions for community acquired pneumonia). Three articles concerned viral airborne diseases, one article about RSV from Norway, one article concerning respiratory tract infections from Finland, and one article about Influenza A and B from Canada). Air temperature and humidity were the two main climatic factors investigated for these diseases. Increased humidity was associated with an increased number of influenza A infections, in contrast decreased humidity and an increased temperature were associated with a decline in influenza B cases (34). An increased temperature was also seen to decrease the risk of pneumonia hospitalization among elderly people (44). Cold temperature and low humidity was associated with increased number of URTI (33). There was no significant association seen between temperature and precipitation 2–3 weeks before ICU admissions for CAP (community acquired pneumonia) (37). For legionellosis, changes in watershed based on hydrological data for Lake Ontario were more important than weather factors (36).

Most of the articles on this disease group were of low to medium quality (Grade 2–3 except for one article about legionellosis given Grade 4). Due to this, the evidence for an association between air temperature, humidity and viral- and bacterial airborne diseases was found to be weak. Even though it seems biologically plausible that climatic factors could be of importance for these diseases, there might be many important co-factors that disguise an association, such as for example human behavior and individual susceptibility. For these diseases, it would have been interesting to study people who are already at risk for respiratory infections, such as people with asthma or COPD, and investigate the additional risk due to certain climatic factors. In some study populations, such as in the paper by (37) on ICU admission due to Community-acquired pneumonia, co-morbidity is likely to be higher among the patients needing ICU care than among the general population. Patients with pulmonary, or cardiac diseases are at higher risk of obtaining severe pneumonia, and are often limited in their daily life due to their diseases, for example in their ability to spend time outside. Thus, the study population in this case should be less likely to be exposed to the weather. It would have been of interest to see studies from rural villages, for example in Greenland, Siberia or Alaska, with an even more extreme climate than in Canada and Scandinavia, and often crowded living conditions.

RSV and influenza are two of the airborne viral diseases, which in studies from outside the Arctic, for example the US, Mexico and Europe, have been most often associated with temperature and humidity (45, 46) but to our knowledge there is not yet enough conclusive evidence for a causal relationship, which is in line with the results in our review. Air temperature and humidity seem to be important climatic factors to investigate further for these diseases.

### Strengths and limitations

To our knowledge, this systematic review is the first in this specific field. Articles were systematically selected and evaluated by two independent readers, and the quality of articles as an indicator for the reliability of results was taken into account when drawing conclusions regarding associations between climatic factors and infectious diseases. The main limitation of this systematic review was that the literature search was only done in PubMed, no other databases or gray literature was considered. We might also have missed some papers because we did not check the references of narrative reviews and did not search in the Web of Knowledge or other databases. On the other hand, most articles concerning human cases of infectious diseases are likely to be available on PubMed. Russian texts were evaluated by one reader only, but discussed with the two others to come to a consensus regarding the quality of the paper. Another limitation was the restriction to studies with human cases of infectious diseases, leaving out animal cases, which might sometimes be the first evidence or indication of a new emerging infectious disease that later can be spread to humans.

## Conclusions

Strong evidence was found in our review for an association between climatic factors and food- and waterborne diseases. Given a well-documented climatic change in the Arctic (1, 2), this emphasizes the need for improved surveillance of these diseases, especially in areas with insufficient infrastructure and therefore a more vulnerable population. More studies are needed to investigate the adverse health impacts of weather and climatic factors in different settings, such as Siberia and other remote communities, but also to confirm a relationship between these factors and vector- and rodent-borne diseases. These studies should also pay attention to confounding and intermediate factors, including environmental characteristics, human behavior and host ecology. Disease and syndromic surveillance implemented at the community level should be part of climate change adaptation measures in the Arctic, as well as monitoring of extreme weather events known to pose a risk for certain infectious diseases.

## References

[1] Pachauri RK, Reisinger A, IPCC, Core Writing Team Climate change 2007: synthesis report. Contribution of Working Groups I, II and III to the Fourth Assessment Report of the Intergovernmental Panel on Climate Change.

[2] ACIA (2004). Impacts of a warming Arctic: Arctic climate impact assessment. ACIA overview report.

[3] Field CB, Barro V, Stocker TF, Qin D, Dokken DJ, Ebi KL, IPCC Glossary of term. Managing the risks of extreme events and disasters to advance climate change adaptation. A Special Report of Working Groups I and II of the Intergovernmental Panel on Climate Change (IPCC).

[4] Evengard B, Sauerborn R (2009). Climate change influences infectious diseases both in the Arctic and the tropics: joining the dots. Glob Health Action.

[5] Parkinson AJ, Butler JC (2005). Potential impacts of climate change on infectious diseases in the Arctic. Int J Circumpolar Health.

[6] Parkinson AJ, Evengard B (2009). Climate change, its impact on human health in the Arctic and the public health response to threats of emerging infectious diseases. Glob Health Action.

[7] Network SIGN (2008). SIGN 50: a guideline developer's handbook revised edition.

[8] Moher D, Liberati A, Tetzlaff J, Altman DG (2009). Preferred reporting items for systematic reviews and meta-analyses: the PRISMA statement. BMJ.

[9] Allard R, Plante C, Garnier C, Kosatsky T (2011). The reported incidence of campylobacteriosis modelled as a function of earlier temperatures and numbers of cases, Montreal, Canada, 1990–2006. Int J Biometeorol.

[10] Auld H, MacIver D, Klaassen J (2004). Heavy rainfall and waterborne disease outbreaks: the Walkerton example. J Toxicol Environ Health A.

[11] Arsenault J, Michel P, Berke O, Ravel A, Gosselin P (2012). Environmental characteristics associated with campylobacteriosis: accounting for the effect of age and season. Epidemiol Infect.

[12] Sandberg M, Nygard K, Meldal H, Valle PS, Kruse H, Skjerve E (2006). Incidence trend and risk factors for campylobacter infections in humans in Norway. BMC Public Health.

[13] Ravel A, Smolina E, Sargeant JM, Cook A, Marshall B, Fleury MD (2010). Seasonality in human salmonellosis: assessment of human activities and chicken contamination as driving factors. Foodborne Pathog Dis.

[14] Fleury M, Charron DF, Holt JD, Allen OB, Maarouf AR (2006). A time series analysis of the relationship of ambient temperature and common bacterial enteric infections in two Canadian provinces. Int J Biometeorol.

[15] Thomas KM, Charron DF, Waltner-Toews D, Schuster C, Maarouf AR, Holt JD (2006). A role of high impact weather events in waterborne disease outbreaks in Canada, 1975–2001. Int J Environ Health Res.

[16] Harper SL, Edge VL, Schuster-Wallace CJ, Berke O, McEwen SA (2011). Weather, water quality and infectious gastrointestinal illness in two Inuit communities in Nunatsiavut, Canada: potential implications for climate change. Ecohealth.

[17] Teschke K, Bellack N, Shen H, Atwater J, Chu R, Koehoorn M (2010). Water and sewage systems, socio-demographics, and duration of residence associated with endemic intestinal infectious diseases: a cohort study. BMC Public Health.

[18] McLaughlin JB, DePaola A, Bopp CA, Martinek KA, Napolilli NP, Allison CG (2005). Outbreak of Vibrio parahaemolyticus gastroenteritis associated with Alaskan oysters. N Engl J Med.

[19] Vasil'ev LV (1970). [Incidence of dysentery and changes in the etiological structure of dysentery in relation to meteorological conditions and number of flies]. Zh Mikrobiol Epidemiol Immunobiol.

[20] Greer AL, Drews SJ, Fisman DN (2009). Why “winter” vomiting disease? Seasonality, hydrology, and Norovirus epidemiology in Toronto, Canada. Ecohealth.

[21] Lindgren E, Gustafson R (2001). Tick-borne encephalitis in Sweden and climate change. Lancet.

[22] Haemig PD, Sjostedt de Luna S, Grafstrom A, Lithner S, Lundkvist A, Waldenstrom J (2011). Forecasting risk of tick-borne encephalitis (TBE): using data from wildlife and climate to predict next year's number of human victims. Scand J Infect Dis.

[23] Tokarevich NK, Tronin AA, Blinova OV, Buzinov RV, Boltenkov VP, Yurasova ED (2011). The impact of climate change on the expansion of *Ixodes persulcatus* habitat and the incidence of tick-borne encephalitis in the north of European Russia. Glob Health Action.

[24] Palo RT (2009). Time series analysis performed on nephropathia epidemica in humans of northern Sweden in relation to bank vole population dynamic and the NAO index. Zoonoses Public Health.

[25] Pettersson L, Boman J, Juto P, Evander M, Ahlm C (2008). Outbreak of Puumala virus infection, Sweden. Emerg Infect Dis.

[26] Ryden P, Bjork R, Schafer ML, Lundstrom JO, Petersen B, Lindblom A (2012). Outbreaks of tularemia in a boreal forest region depends on mosquito prevalence. J Infect Dis.

[27] Bennet L, Halling A, Berglund J (2006). Increased incidence of Lyme borreliosis in southern Sweden following mild winters and during warm, humid summers. Eur J Clin Microbiol Infect Dis.

[28] Hulden L, Hulden L, Heliovaara K (2005). Endemic malaria: an ‘indoor’ disease in northern Europe. Historical data analysed. Malar J.

[29] Hulden L, Hulden L (2009). The decline of malaria in Finland – the impact of the vector and social variables. Malar J.

[30] Tizard IR, Fish A, Quinn JP (1976). Some observations on the epidemiology of toxoplasmosis in Canada. J Hyg (Lond).

[31] Brummer-Korvenkontio M, Vapalahti O, Kuusisto P, Saikku P, Manni T, Koskela P (2002). Epidemiology of Sindbis virus infections in Finland 1981–96: possible factors explaining a peculiar disease pattern. Epidemiol Infect.

[32] Orstavik I, Carlsen KH, Halvorsen K (1980). Respiratory syncytial virus infections in Oslo 1972–1978. I. Virological and epidemiological studies. Acta Paediatr Scand.

[33] Mäkinen TM, Juvonen R, Jokelainen J, Harju TH, Peitso A, Bloigu A (2009). Cold temperature and low humidity are associated with increased occurrence of respiratory tract infections. Respir Med.

[34] Tang JW, Lai FY, Nymadawa P, Deng YM, Ratnamohan M, Petric M (2010). Comparison of the incidence of influenza in relation to climate factors during 2000–2007 in five countries. J Med Virol.

[35] Crighton EJ, Elliott SJ, Moineddin R, Kanaroglou P, Upshur R (2007). A spatial analysis of the determinants of pneumonia and influenza hospitalizations in Ontario (1992–2001). Soc Sci Med.

[36] Ng V, Tang P, Jamieson F, Drews SJ, Brown S, Low DE (2008). Going with the flow: legionellosis risk in Toronto, Canada is strongly associated with local watershed hydrology. Ecohealth.

[37] Dodek PM, Norena M, Keenan SP, Teja A, Wong H (2011). Intensive care unit admissions for community-acquired pneumonia are seasonal but are not associated with weather or reports of influenza-like illness in the community. J Crit Care.

[38] D'Souza RM, Becker NG, Hall G, Moodie KB (2004). Does ambient temperature affect foodborne disease?. Epidemiology.

[39] Kovats RS, Edwards SJ, Charron D, Cowden J, D'Souza RM, Ebi KL (2005). Climate variability and campylobacter infection: an international study. Int J Biometeorol.

[40] Lake IR, Gillespie IA, Bentham G, Nichols GL, Lane C, Adak GK (2009). A re-evaluation of the impact of temperature and climate change on foodborne illness. Epidemiol Infect.

[41] Cann KF, Thomas DR, Salmon RL, Wyn-Jones AP, Kay D (2013). Extreme water-related weather events and waterborne disease. Epidemiol Infect.

[42] Gage KL, Burkot TR, Eisen RJ, Hayes EB (2008). Climate and vectorborne diseases. Am J Prev Med.

[43] du Prel JB, Puppe W, Grondahl B, Knuf M, Weigl JA, Schaaff F (2009). Are meteorological parameters associated with acute respiratory tract infections?. Clin Infect Dis.

[44] Crighton EJ, Moineddin R, Mamdani M, Upshur RE (2004). Influenza and pneumonia hospitalizations in Ontario: a time-series analysis. Epidemiol Infect.

[45] Yusuf S, Piedimonte G, Auais A, Demmler G, Krishnan S, Van Caeseele P (2007). The relationship of meteorological conditions to the epidemic activity of respiratory syncytial virus. Epidemiol Infect.

[46] Viboud C, Pakdaman K, Boelle PY, Wilson ML, Myers MF, Valleron AJ (2004). Association of influenza epidemics with global climate variability. Eur J Epidemiol.

